# Nitrite isotope characteristics and associated soil N transformations

**DOI:** 10.1038/s41598-021-83786-w

**Published:** 2021-03-03

**Authors:** Dominika Lewicka-Szczebak, Anne Jansen-Willems, Christoph Müller, Jens Dyckmans, Reinhard Well

**Affiliations:** 1 Centre for Stable Isotope Research and Analysis, University of Göttingen, Göttingen, Germany; 2 Laboratory of Isotope Geology and Geoecology, Institute of Geological Sciences, University of Wrocław, Wrocław, Poland; 3 Institute of Plant Ecology, Justus Liebig University, Giessen, Germany; 4 School of Biology and Environmental Science and Earth Institute, University College Dublin, Belfield, Dublin, Ireland; 5 Thünen-Institut of Climate-Smart Agriculture, Braunschweig, Germany

**Keywords:** Biogeochemistry, Environmental sciences

## Abstract

Nitrite (NO_2_^−^) is a crucial compound in the N soil cycle. As an intermediate of nearly all N transformations, its isotopic signature may provide precious information on the active pathways and processes. NO_2_^−^ analyses have already been applied in ^15^N tracing studies, increasing their interpretation perspectives. Natural abundance NO_2_^−^ isotope studies in soils were so far not applied and this study aims at testing if such analyses are useful in tracing the soil N cycle. We conducted laboratory soil incubations with parallel natural abundance and ^15^N treatments, accompanied by isotopic analyses of soil N compounds (NO_3_^−^, NO_2_^−^, NH_4_^+^). The double ^15^N tracing method was used as a reference method for estimations of N transformation processes based on natural abundance nitrite dynamics. We obtained a very good agreement between the results from nitrite isotope model proposed here and the ^15^N tracing approach. Natural abundance nitrite isotope studies are a promising tool to our understanding of soil N cycling.

## Introduction

Nitrite (NO_2_^−^), as an intermediate of nearly all N transformations, is a crucial compound to understand the complexity of the N soil cycle with its many contributing pathways. Moreover, as a very reactive compound it usually occurs at very low concentrations, hence conveying information on currently active N transformations. The *Ntrace* model used for interpretation of ^15^N labelled soil studies has been recently expanded with the NO_2_^−^ content and isotopic analyses, which vastly increased its interpretation perspectives^1^. Thanks to incorporation of NO_2_^−^ dynamics in this model it appeared possible to distinguish and quantify three NO_2_^−^ and N_2_O production pathways: denitrification, autotrophic nitrification and heterotrophic nitrification. Although ^15^N tracing studies can precisely identify various soil N transformations^1,2^, they require addition of ^15^N -labelled substances, which is associated with additional fertilization, soil disturbance, and potential problems with label distribution homogeneity^3,4^. Moreover, due to high costs and fast consumption of the ^15^N label, ^15^N tracing approach can be applied mostly for short-term and micro-plot studies^5^. Development of reliable methods for identifying N transformations based on natural abundance stable isotopes can overcome these problems and provide an approach allowing studies in undisturbed soil conditions ensuring original N transformation rates that can be traced in larger time and space scale.

Natural abundance NO_2_^–^isotope studies are so far mostly applied in aquatic studies^6–9^ and appeared particularly informative for the oceanic oxygen deficient zones, where NO_2_^−^ can be accumulated^7,9^. However, for soil studies the natural abundance NO_2_^−^ analyses are so far lacking. Also in soils NO_2_^−^ accumulation may happen and the monitoring of NO_2_^−^ content in soils can provide important information to understand the N cycle^10–12^. In particular, NO_2_^−^ plays a central role for N_2_O formation^12,13^. However, even in situations when NO_2_^−^ accumulation is not observed, and the interpretation of, typically very low, soil NO_2_^−^-contents is ambiguous, the N transformations can potentially be followed by the stable isotopic signature of NO_2_^−^, which has neither been tested nor applied so far.

Nitrite can be formed during nitrate reduction (*NAR*) in the course of denitrification, ammonium oxidation (*AOX*) in the course of autotrophic nitrification and organic N oxidation (*ORG*) associated with heterotrophic nitrification, and consumed during nitrite reduction (*NIR*) to NO or N_2_O, and nitrite oxidation (*NIOX*) to NO_3_^−^^1,7^. Each of these sources and sinks are characterised by specific isotopic fractionation^7,9,14^, which makes it possible to trace them back to their origins and sinks of NO_2_^−^, and consequently, for a better understanding of the N cycling^7^.

This study presents the first attempt to interpret the NO_2_^−^ isotopic signatures (δ^15^N_NO2−_ and δ^18^O_NO2−_) in agricultural soil to decipher soil transformation processes. Three laboratory incubations were performed: under oxic conditions with lower water content (L1), under oxic conditions with higher water content (L2) and under anoxic conditions (L3), to monitor the differences when various N transformation processes are enhanced. The incubations at natural abundance level (NA treatment) and under ^15^N enrichment (^15^NO_3_ treatment and ^15^NH_4_ treatment) were performed simultaneously. Based on the ^15^N treatments the *Ntrace* model^1^ was applied to determine NO_2_^−^ sources and sinks. The results of NA treatment were used to construct the soil NO_2_^−^ model, which is based on the model used for oceanic studies^7^, including the processes that have contributed to production and consumption of NO_2_^−^ in soils. This study provides the first attempt to validate the results of NO_2_^−^ isotope modelling with an independent ^15^N tracing approach.

## Results

### Soil NO_2_^−^ characteristics

**Figure 1:**
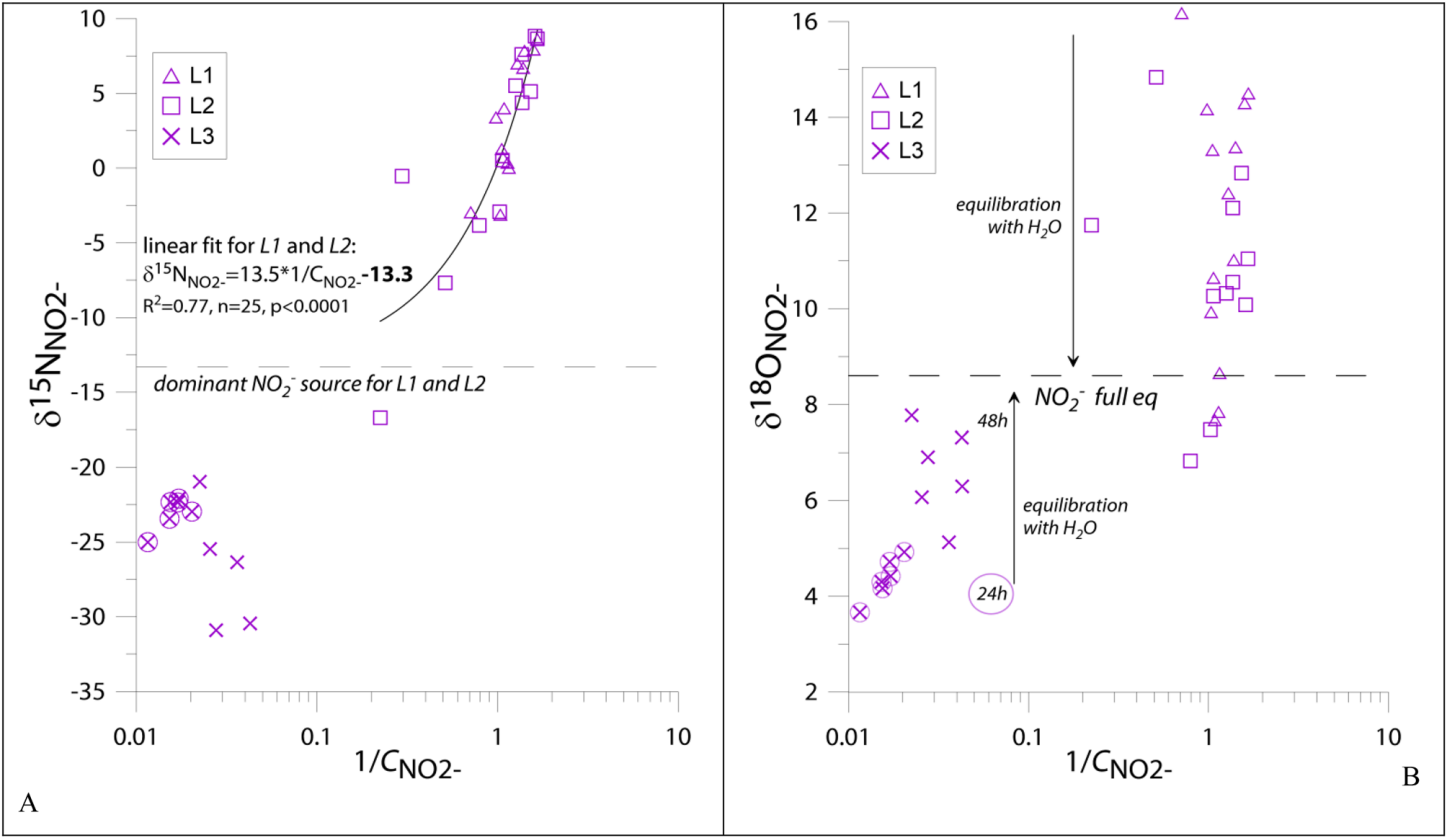
Relationship between NO_2_^−^ isotopic signature δ^15^N_NO2−_ (**A**) or δ^18^O_NO2−_ (**B**) and reciprocal NO_2_^−^ content (Keeling plot analysis—see “Methods” section). For δ^18^O_NO2−_ (**B**) the dashed line indicates the δ value of NO_2_^−^ in full equilibrium with ambient water in 20 °C of 8.6‰^23^ and the arrows indicate the direction of change in δ values of NO_2_^−^in course of equilibration with water (points ‘move’ towards full equilibrium). For L3 the first samples taken after 24 h are marked with circles and the second samples taken after 48 h are shown with crosses only. Note the logarithmic scale of the X-axis.

The oxic experiment was performed in two moisture treatments: L1 (dryer conditions) and L2 (wetter conditions), with water addition in the middle of experiment which increased the soil moisture from 61 to 68% water-filled pores space (WFPS) for L1a and L1b and from 72 to 81% WFPS for L2a and L2b, respectively. The detailed experimental conditions and information on general soil properties can be found in^15^ and in the supplement. NO_2_^−^ content varied from 0.6 to 1.4 μmol N kg^−1^ soil for L1 and from 0.1 to 4.7 for L2, whereas the NO_3_^−^ content was three orders higher and quite stable ranging from 1300 to 1700 μmol N kg^−1^ soil. The δ^15^N_NO2−_ was similar for L1 and L2 with a mean of 3.2 ± 4.2‰ and 3.9 ± 4.2‰, respectively, whereas δ^15^N_NO3−_ was very stable with a mean of 4.5 ± 0.4‰ and 4.7 ± 0.6‰, respectively. There was a negative correlation between δ^15^N_NO2−_ and the NO_2_^−^-content (Fig. 1A). Similar values for δ^18^O_NO2−_ were found for both L1 and L2 with a mean of 11.8 ± 2.8‰ and 12.5 ± 5.0‰, respectively.

The anoxic experiment L3 was performed to favour denitrification. NO_2_^−^ content was much higher when compared to oxic conditions (L1 and L2) reaching 63.9 ± 12.5 μmol N kg^−1^ soil and 32.4 ± 8.9 μmol kg^−1^ soil after 24 h and 48 h of incubation, respectively. The average δ^15^N_NO2−_ was − 24.8 ± 3.3‰ without significant differences between the two samplings, whereas δ^18^O_NO2−_ showed significantly lower values of 4.4 ± 0.4‰ after 24 h compared to 6.6 ± 1.0‰ after 48 h (Fig. 1B).

In both ^15^N treatments (^15^NH_4_^+^ and ^15^NO_3_^−^) in L1 and L2, we observed a sudden drop in ^15^N abundance in NO_2_^−^ (*a*^15^N_NO2−_) from 12.7 to 5.1 at.% after water addition to the soil, whereas ^15^N abundance in NO_3_^−^ (*a*^15^N_NO3−_) showed only slight decrease from 13.2 to 12.1 at.% (means for all treatments, individual values in Fig. S1 and Table S1). This indicates an incorporation of another source of unlabelled NO_2_^−^ for the wet part of these experiments. In natural abundance isotopes this change was also reflected in a higher apparent isotope effect ^15^*η*_NO2−/ NO3−_. For the wet part it was even positive with an average + 1.6‰, whereas for the dry part it was lower with − 4.4‰ (Table 1). Interestingly, this significant isotopic change in NO_2_^−^ was not reflected in the N_2_O ^15^N abundance (*a*^15^N_N2O_) in the ^15^N treatments (Fig. S1, Table S1).

**Table 1:** Apparent isotopic fractionation factors for *δ*^15^N of NO_3_^−^, NO_2_^−^ and N_2_O.

Experiment	^15^*η*_NO2–NO3_	^15^*η*_N2O–NO3_	^15^*η*_N2O–NO2_
L1a	− 5.0 ± 1.9	− 23.5 ± 1.9	− 18.5 ± 1.8
L1b	1.8 ± 1.8	− 15.0 ± 9.3	− 16.6 ± 7.4
L2a	− 3.8 ± 5.2	− 22.7 ± 5.9	− 18.1 ± 11.2
L2b	1.4 ± 2.8	− 41.6 ± 2.1	− 42.8 ± 0.3
L3	− 31.4 ± 4.3	− 53.1 ± 2.7	− 21.3 ± 2.9

### Isotope effects between NO_3_^−^, NO_2_^−^and N_2_O

With the NA dataset for NO_2_^−^ presented in this paper and the dataset for N_2_O presented in a previous paper for L1 and L2^15^, and here for L3 (Table S1), we can investigate the relation between the isotopic characteristics of both N compounds and determine the apparent isotope effects between NO_2_^−^ and N_2_O, and comparing them with isotope effects between NO_3_^−^ and N_2_O. Therefore, we need the isotopic signatures of the produced N_2_O prior to isotopic fractionation due to N_2_O reduction. For L3 the incubations were partially conducted with N_2_O reduction inhibition (acetylated treatments) and we only report here the δ_N2O_ values of the inhibited treatment (Table S1) which represent the produced N_2_O isotopic signatures. For L1 and L2 a detailed study of N_2_O reduction was performed^15^ where the N_2_O reduced fraction (*r*_N2O_) was determined with ^15^N treatment, and the produced N_2_O (*δ*_N2O_p_) can be calculated according to the equation:

$${\delta }_{N2O\_p}={\delta }_{N2{O}_{m}}-\mathrm{ln}{r}_{N2O}*{\varepsilon }_{red}$$

based on the measured N_2_O (*δ*_N2O_m_) and the isotopic fractionation associated with N_2_O reduction (*ε*_red_)^15^. The determined apparent N isotope effects (^15^*η*) was calculated as:

$${{}^{15}\eta }_{product-substrate}={\delta }^{15}{N}_{product}-{\delta }^{15}{N}_{substrate}$$

### Determination of NO_2_^−^ dominant source

Keeling plots were applied to identify the NO_2_^−^ dominant source (see Methods Section for methodical explanation)^15–18^. For the oxic experiment, a significant linear fit between δ^15^N_NO2−_ and reciprocal NO_2_^−^ content was found, where an linear equation intercept of − 13.3‰ indicated the isotopic signature of the dominant NO_2_^−^ source. It must be denitrification since the applied conditions of quite high soil moisture and nitrate amendment should have favoured denitrification. In a previous study, denitrification was identified as the dominant source for N_2_O^15^ and also the applied *Ntrace* model indicated the dominance of denitrification nitrate reduction (*NAR*) in the NO_2_^−^-sources (*f*_*NAR*_ of 0.53 and 0.55 for L1 and L2, respectively, Table 2). Hence, based on the value found from the Keeling plot (Fig. 1A) we can determine the nitrogen isotopic fractionation for denitrification (^15^*ε*_NAR_) between *δ*^15^N_NO3−_ (mean measured value) and *δ*^15^N_NO2−_ (Keeling plot intercept) for this incubation experiment:

$$^{{{15}}} \varepsilon_{NAR} \left( {{\text{NO}}_{{2}}^{ - } /{\text{ NO}}_{{3}}^{ - } } \right) = - {13}.{3}\textperthousand{-}\left( { + {4}.{5}\textperthousand} \right) = - {17}.{8}\textperthousand $$

This value fits quite well in the literature range^7,14^ and is further used in the NO_2_^−^ isotope model as ^15^*ε*_NAR_.

**Table 2:** Nitrite stable isotope model to determine sources mixing proportions.

	Source	Substrate		Source fractionation		Produced NO_2_^−^		***f***_**mix**_	Mixed NO_2_^−^		Sink	Sink fractionation		Residual NO_2_^−^		NO_2_^−^ eq	*f*_red-ox_	Final modeled		True measured		***f***_**mix**_
		*δ*^18^O	*δ*^15^N	^18^ε	^15^ε	*δ*^18^O	*δ*^15^N	***N***_***trace***_	*δ*^18^O	*δ*^15^N		^18^ε	^15^ε	*δ*^18^O	*δ*^15^N	*δ*^18^O	*N*_*trace*_	*δ*^18^O	*δ*^15^N	*δ*^18^O	*δ*^15^N	***fitted***
L1	*NAR*	4.3	4.5	0	− 17.8	4.3	− 13.3	**0.53**	10.9	0.3	*NIR*	− 4.0	− 10.0	14.9	10.3	13.3	0.86	12.4	7.1	11.8	3.2	**0.55**
	*AOX*	23.5; − 6.4	93.9	20	− 25.0	18.4	68.9	**0.08**			*NIOX*	5.0	13.0	5.9	− 12.7	6.6	0.14					**0.02**
	*ORG*	23.5	7.4	0	− 2.0	18.4	5.4	**0.39**														**0.43**
L2	*NAR*	4.7	4.7	0	− 17.8	4.7	− 13.1	**0.55**	10.9	3.2	*NIR*	− 4.0	− 10.0	14.9	13.2	13.3	0.70	11.3	6.3	12.5	3.9	**0.58**
	*AOX*	23.5; − 6.4	65.5	− 20	− 25.0	18.4	40.5	**0.23**			*NIOX*	5.0	13.0	5.9	− 9.8	6.6	0.30					**0.18**
	*ORG*	23.5	7.4	0	− 2.0	18.4	5.4	**0.23**														**0.25**
L3	*NAR*	15.3	7.0	− 10	− 30	5.3	− 23.0	**1**	5.3	− 23.0	*NIR*	− 4.0	− 10.0	9.3	− 13.0	9.1		9.1	− 13.0	5.5	− 24.8	
											*no fract*			5.3	− 23.0	6.1		6.1	− 23.0			

In the first step, nitrite isotopic signature (*δ*^18^O, *δ*^15^N) is modelled based on: (i) the nitrite sources taking into account measured substrate isotopic signatures (NO_3_^−^ for *NAR*, NH_4_^+^ for *AOX* and organic N for *ORG*), fractionation factors (^18^*ε*, ^15^*ε*), and sources mixing proportions according to the results of the *N*_*trace*_ model (*f*_mix_ *N*_*trace*_); (ii) nitrite sinks with their characteristic isotopic fractionation factors (^18^*ε*, ^15^*ε*) including the nitrite reduction–oxidation ratio after results of the *N*_*trace*_ model; and (iii) nitrite equilibration with water (NO_2_^−^ eq) including measured extent of O-exchange of 0.25, *δ*^18^O of -5‰ and ^18^ε_eq_ for 20 °C. In the second step, modelled nitrite isotopic signature were fitted to the measured values by adjusting the sources mixing proportions (*f*_mix_ *fitted*) to find the ideal fit of modelled vs. measured *δ*^15^N values.

Under anoxic conditions, denitrification should be the only source of NO_2_^−^, hence a typical Keeling correlation is not expected. We rather observed the opposite trend in L3 than under oxic conditions, i.e. lower δ^15^N_NO2−_ values with lower NO_2_^−^ contents (Fig. 1A). Most probably this reflects the variability of apparent isotope effects, which are typically larger for lower reaction rates^19,20^.

For δ^18^O_NO2−_ values, beside sources mixing, we also deal with isotope exchange of O-atoms between NO_2_^−^ and ambient water, hence the Keeling plot method cannot be applied. We observed that δ^18^O_NO2−_ values were modified by the O exchange process, especially under anoxic conditions (L3), where lower NO_2_^−^ content and incubation progress shifted δ^18^O_NO2−_ values towards equilibrium with water (NO_2_^−^ full eq, Fig. 1B). NO_2_^−^ samples taken after 48 h showed more equilibrated δ^18^O_NO2−_ values and lower NO_2_^−^ content.

### NO_2_^−^ isotope model

**Figure 2:**
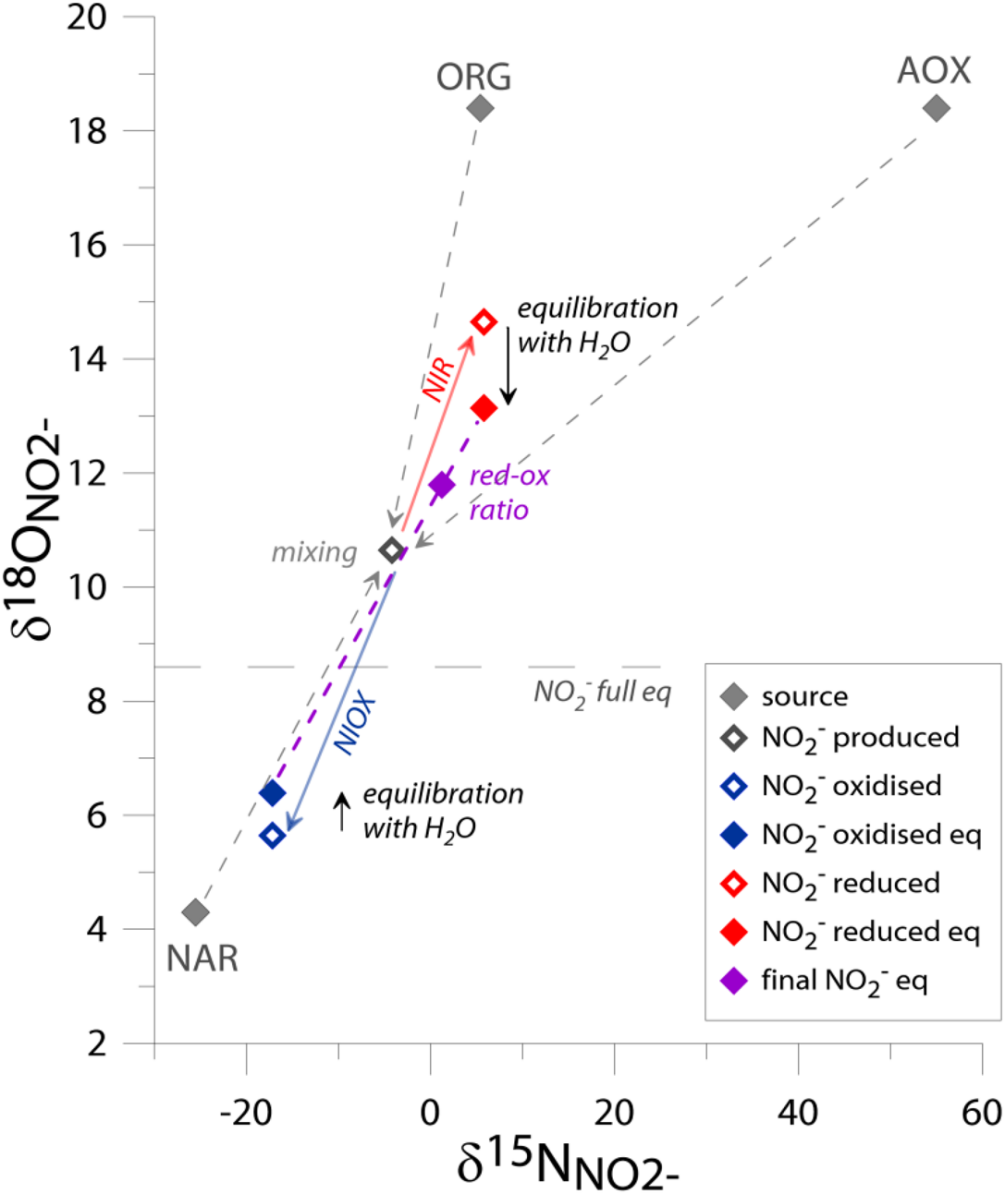
General scheme of the NO_2_^−^ stable isotope model. The isotopic signatures of NO_2_^−^ sources shown are based on the measured mean isotopic signatures of substrates for L1 and L2 and the isotopic fractionation associated with NAR, AOX and ORG (Table 1). Dashed gray arrows illustrate the mixing of 3 NO_2_^−^ sources with mean mixing proportions found in *Ntrace* study (*f*_NAR_ = 0.55, *f*_AOX_ = 0.15, *f*_ORG_ = 0.30, Table 1) resulting in the produced *δ*_NO2_- (grey open point). This *δ* value can be modified by NIR (red arrow) and NIOX (blue arrow). The δ^18^O_NO2_- after reduction or oxidation (red and blue open point, respectively) is further modified by equilibration with ambient water with the extent of 0.25 of the equilibrated oxygen atoms (red and blue filled point, respectively). The ratio of NO_2_- reduction and oxidation processes (red-ox ratio, here 4:1, as mean from Ntrace study, Table 1) determines the final δ_NO2_- (purple point).

The model is constructed based on the NO_2_^−^ isotope model proposed for oceanic studies^7^ and adapted for typical soil N pathways after the *Ntrace* model, designed for ^15^N labelled soil studies applying NO_2_^−^ as a key intermediate in soil N transformations^1^, assuming steady state conditions. It takes into account three main NO_2_^−^ sources (*NAR*, *AOX* and *ORG*) and two main NO_2_^−^ sinks (*NIR* and *NIOX*), as well as δ^18^O_NO2−_ equilibration with ambient water (Fig. 2), according to the following equations:

1 $${\delta }^{15}{N}_{NO2-}={\delta }^{15}{N}_{NAR}*{f}_{NAR}+{\delta }^{15}{N}_{AOX}*{f}_{AOX}+{\delta }^{15}{O}_{ORG}*{f}_{ORG}-{{}^{15}\varepsilon }_{NIR}*{f}_{NIR}-{{}^{15}\varepsilon }_{NIOX}*{f}_{NIOX}$$

2 $${\delta }^{18}{O}_{NO2-}={(\delta }^{18}{O}_{NAR}*{f}_{NAR}+{\delta }^{18}{O}_{AOX}*{f}_{AOX}+{\delta }^{18}{O}_{ORG}*{f}_{ORG}-{{}^{18}\varepsilon }_{NIR}*{f}_{NIR}-{{}^{18}\varepsilon }_{NIOX}*{f}_{NIOX})*\left(1-x\right)+{\delta }^{18}{O}_{eq}*x$$

where δ_NO2−_ is the measured residual NO_2_^−^ isotopic signature, δ_NAR/AOX/ORG_ are the isotopic signatures of source NO_2_^−^ calculated with the measured stable isotope values for NO_2_^−^ substrates (NO_3_^−^, NH_4_^+^, Norg, respectively for three sources, Table 2) and the characteristic isotopic fractionation associated with each NO_2_^−^ formation pathway (ε_NAR/AOX/ORG_, Table 2). ε_NIR/NIOX_ are the isotopic fractionation factors associated with NO_2_^−^ sinks (ε_NIR/NIOX_, Table 2). See also Methods Section for detailed description of isotope effects for particular processes; final values used in the model are shown in Table 2. The δ^18^O_eq_ stands for O isotopic signature of NO_2_^−^ in complete equilibrium with water, which equals 8.6‰ for the incubation temperature of 20 °C^7^ and δ^18^O_H2O_ of − 5‰. *x* is the extent of oxygen atom exchange between nitrate and ambient water determined with the ^17^O approach^21^ for N_2_O originating from denitrification processes under anoxic conditions (L3) and is equal 0.25 (see Methods Section). The exchange for NO_2_^−^ cannot be higher than the value determined for N_2_O. Since most of the exchange observed for N_2_O is associated with the NO_2_^—^-H_2_O isotope exchange^21^ this value was incorporated in the model calculations.

For NAR we were able to determine the isotope effect (^15^*ε*_NAR_ =  − 17.8) based on the Keeling plot (Fig. 1A) for L1 and L2. This value of − 17.8‰ is on the lower range of previously determined values^22–29^ (see summary in the Methods Section) and was used in the model for L1 and L2. For anoxic experiment L3 the fractionation was larger (Fig. 1A) and we included the typical ^15^ε_NAR_ value of − 30‰ in the model. For ^18^ε_NAR_ the fractionation must be very low to obtain the observed range of δ^18^O_NO2−_: − 10‰ for L3 and no fractionation for L1 and L2. Similar ranges of ^18^ε_NAR_ values were modelled previously with indication of lower values for smaller reaction rates^21^. This is in accordance with our observations indicating much lower N transformation rates for the oxic experiments L1 and L2 than for the anoxic L3. Also the isotopic fractionation for NIR appears to be lower under anoxic conditions, where NO_2_^−^ is accumulating. We obtained best fit between modelled and measured values for L3 when no fractionation associated with NIR was assumed (Table 2). This can be due to observed accumulation of NO_2_^−^, indicating that the steady-state model assumption is not valid. In case of NO_2_^−^ accumulation the isotopic fractionation associated with its reduction has a very low impact on the final isotopic signature of the residual NO_2_^−^.

### NO_2_^−^ turnover

When *δ*^18^O_NO2-_ values are not completely equilibrated with soil water, measured δ^18^O_NO2−_ values can be used to estimate the rates of biological NO_2_^−^ turnover relative to abiotic exchange^7^. This estimation is based on the abiotic equilibration rate as a function of temperature and pH^7^. Furthermore, we can determine the flux of NO_2_^−^ oxygen atoms abiotic exchange as *F*_eq_ = k*C_NO2−_. The NO_2_^−^ flux of biological production (or consumption) can be determined from the δ^18^O_NO2-_ isotope mass balance following the method proposed for oceanic studies^7^ adapted to soil NO_2_^−^ transformations:

3 $${F}_{B}=\frac{{F}_{eq}({ \delta }^{18}{O}_{NO2-}-{\delta }^{18}{O}_{eq})}{{\delta }^{18}{O}_{NAR}*{f}_{NAR}+{\delta }^{18}{O}_{AOX}*{f}_{AOX}+{\delta }^{18}{O}_{ORG}*{f}_{ORG}-{{}^{18}\varepsilon }_{NIR}*{f}_{NIR}-{{}^{18}\varepsilon }_{NIOX}*{f}_{NIOX}-{ \delta }^{18}{O}_{NO2-}}$$

where *δ*^18^O_NO2-_ is the measured NO_2_^−^ , *δ*^18^O_NAR/AOX/ORG_ are the calculated NO_2_^−^ sources NAR, AOX and ORG, ^18^ε_NIR/NIOX_ are the isotopic fractionation associated with NO_2_^−^ sinks NIR and NIOX, *f* are the respective contributions of NO_2_^−^ sources determined by *Ntrace* model (Table 2), and *δ*^18^O_eq_ is the value for NO_2_^−^ in complete equilibrium with ambient water (of 8.6‰ for this case study). In turnover rate calculations (Table 3) we have neglected NIOX because due to the inverse fractionation of this process for some cases the isotope mass balance did not work due to unrealistic discrepancies between calculated and measured δ^18^O_NO2−_ values. Since ^18^ε_NIOX_ can be very low^30^ and the NIOX contribution is low for our case study (up to 30%, Table 2), this process has most probably little impact on the final δ^18^O_NO2−_ values. In the NO_2_^−^ isotope model, neglecting NIOX would result in higher final modelled *δ*^18^O_NO2_ values of 13.3‰ for both treatments, which would fit to the measured values equally well as when the NIOX fractionation is included (Table 2).

**Table 3:** Nitrite transformation fluxes (due to equilibration (*F*_eq_) and biological turnover (*F*_B_)) and residence time due to biological turnover (*Γ*_B_) or abiotic equilibration (*Γ*_eq_) determined with δ^18^O_NO2_- values compared to nitrite turnover rate determined with *Ntrace* model (*F*_*Ntrace*_).

	*k*	*C*_NO2−_	*F*_eq_	*F*_B_	*F*_*Ntrace*_	*Γ*_B_	*Γ*_eq_
	[d^−1^]	[μmol kg^−1^]	[μmol kg^−1^ d^−1^]	[μmol kg^−1^ d^−1^]	[μmol kg^−1^ d^−1^]	[h]	[h]
L1	17.7	0.9	15.5	**16.3**	**28.0**	1.3	1.4
L2	17.7	1.4	24.3	**39.6**	**26.4**	0.8	1.4
L3	17.7	48.1	851.8	639.7		1.8	1.4

Our results indicate that the NO_2_^−^ flux in L2 is larger than in L1 (Table 3), which is reasonable since L2 was the wetter treatment showing more intensive nitrogen fluxes based N_2_O and N_2_ fluxes, which were twice as high in L2 compared to L1^15^. Similar differences can be observed here for calculated FB values (Table 3), however this is not directly confirmed by the *Ntrace* results.

## Discussion

We found very good congruity between *Ntrace* and the NA NO_2_^−^ model. The modelled δ^18^O_NO2−_ and δ^15^N_NO2−_ values using measured source fractions provided by the *Ntrace* model differed up to 1.2‰ and 4.0‰, respectively, when compared to true measured values (Table 2). When we solely used the NA NO_2_^−^ model to assess the fraction of NO_2_^−^-sources contribution based on δ^15^N_NO2−_, i.e., fitting modelled δ^15^N_NO2−_ values to the true measured values by adjusting the fraction of NO_2_^−^-sources contribution, the fitted fractions are in good agreement with fractions provided by the *Ntrace* model (Table 2). Both results show similar dominance of NAR in NO_2_^−^- production (*f*_NAR_ of ca. 0.55) but the NA NO_2_^−^ model indicates even higher contribution of heterotrophic vs autotrophic nitrification (*f*_ORG_ vs. *f*_AOX_).

The *Ntrace* approach, which is able to identify the contribution of ORG, was actually the first one that paid attention to this process in soils^31^. Here, with the NA NO_2_^−^ model we get a confirmation of the potentially high ORG relevance in soil N transformations. Without this process the final δ^15^N_NO2−_ and δ^18^O_NO2−_ values could not be explained. Namely, if only considering two source processes: NAR and AOX, to meet the measured δ^15^N_NO2−_ value we would need domination of NAR (*f*_NAR_ > 0.75) and to meet the measured δ^18^O_NO2−_ value we would need an unrealistically high contribution of AOX (*f*_AOX_ > 0.70). Hence, the application of both isotope signatures (δ^15^N_NO2−_ and δ^18^O_NO2−_) simultaneously allows for a proper identification of NO_2_^−^ sources.

The presented NO_2_^−^ isotope model may not be very typical, since for our case study we had exceptionally high δ^15^N_NH4+_ values (from 36 to 100‰), hence this worked partially as a naturally low level ^15^N tracing allowing for very clear separation of NAR and AOX with δ^15^N_NO2−_ values. In case of similar δ values for substrate NO_3_^−^ and NH_4_^+^, this separation would be very weak, but still, in combination with δ^18^O_NO2−_ values, may be useful in assessing source contributions. The ^15^N enrichment of NH_4_^+^ was not purposely induced but was a consequence of the fast ammonium consumption. *Ntrace* analysis revealed that the dominant ammonium sink is immobilisation, responsible for more than 90% of ammonium consumption. This process is associated with pronounced enrichment of residual ammonium in ^15^N^32–34^, which we observed in this study. The very fast NH_4_^+^ immobilisation and its further release due to Norg oxidation to NO_3_^−^ were unexpected in this study and cannot be fully explained. The *Ntrace* model assumes the existence of the labile Norg pool, which is associated with these extremely fast fluxes. In the NA model, the assumed substrate for ORG nitrite production is the measured δ^15^N of the bulk organic N pool. This is probably the largest uncertainty in the model, since the labile Norg pool may be isotopically different than the bulk Norg. Similar uncertainties may also be associated with the measured bulk δ^15^N_NO3−_, since this value may be significantly higher in the intensively denitrifying soil microsites.

The NO_2_^−^ isotopic signature time series in the ^15^N treatments (Fig. S1), strongly indicates the appearance of new unlabelled NO_2_^−^ for L1 and L2 after water addition in the course of the incubation. *Ntrace* clearly indicated an increase in ORG contribution after water addition (from 0.11 to 0.49 and from 0.07 to 0.33 for L1 and L2, respectively, Table S3) and the NA NO_2_^−^ model confirms this finding (from 0.11 to 0.52 and from 0.21 to 0.31 for L1 and L2, respectively, Table S3). This suggests that NA NO_2_^−^ analyses can be used to trace the dynamic changes in soil N transformations.

The ^15^N treatment indicated that N_2_O ^15^N enrichment (*a*^15^N_N2O_) follows rather *a*^15^N_NO3−_ than *a*^15^N_NO2−_ (Fig. S1). This indicates that mostly NAR NO_2_^−^ is further reduced and emitted as N_2_O and suggests that the ORG NO_2_^−^ forms an isolated NO_2_^−^ pool, as also suggested earlier^1^, which is probably not further reduced to N_2_O in significant magnitude. The calculated *a*_P_N2O_ value representing the ^15^N enrichment of the ^15^N -pool derived N_2_O is higher than *a*^15^N_N2O_ due to the contribution of non-labelled N_2_O to the total N_2_O flux, so that *a*^15^N_N2O_ = *f*_*P_*N2O_ * *a*_*P_*N2O_ + (1- *f*_*P_*N2O_ ) * *a*_NA_ , where *a*_NA_ is the ^15^N abundance of the natural abundance samples (0.367 at.%). However, *a*_*P_*N2O_ values always have a higher ^15^N abundance than found for any soil N-pool (Table S1). This indicates that in denitrification soil microsites, where the ^15^N-pool derived N_2_O is produced, we deal with higher *a*^15^N_NO2−_ and *a*^15^N_NO3−_ values than the mean analysed values. This confirms the N_2_O emission originating from various isolated soil N-pools. The fraction of the ^15^N -pool N_2_O is dominating – from 0.7 to 1.0—with higher values for higher soil moisture (Table S1). This is in contrast to NO_2_^−^ which gets ^15^N depleted after water addition (Fig. S1) and f_NAR_ is only around 0.5.

Regarding the NA isotope effects, we can see that for pure denitrification processes under anoxic conditions in L3, we deal with very low ^15^*η*_NO2−NO3_ values (Table 1) indicating a pronounced isotope effect between NO_3_^−^ and NO_2_^−^, whereas for L1 and L2, where NO_2_^−^ is formed not only due to NAR but also AOX and ORG (Table S2), this effect is much smaller (as indicated by ^15^*η*_NO2−NO3_ closer to 0, Table 1). Interestingly, in the wetter parts of the experiments (L1b, L2b), when increased contribution of ORG NO_2_^−^ occurs, even an inverse effect is observed, i.e. NO_2_^−^ is ^15^N enriched compared to NO_3_^−^. As a result, for L1 and L2 very similar isotope effects for N_2_O production with both substrates are observed (^15^*η*_N2O-NO3_ and ^15^*η*_N2O-NO2_ are not significantly different). This is in contrast with L3, where ^15^*η*_N2O-NO2_ is much lower compared to ^15^*η*_N2O-NO3_. For oxic conditions, clearly the highest isotope effect for N_2_O production with both substrates is noted for L2b, for which the ^15^*η*_N2O-NO3_ is nearest to the values typical for denitrification, as found in L3. Indeed, for L2b the denitrification pool derived fraction (*f*_P_N2O_, Table S1) is the highest. Also the previous study^15^ indicated that the N_2_O fraction produced due to denitrification, including both bacterial and fungal denitrification, is near 1 for this part of the experiment (L2b)^15^. However, the ^15^*η*_N2O-NO2_ values are much lower for L2b than for L3 which is probably caused by admixture of other nitrite sources present for L2b but absent for L3.

Despite the fact that NO_2_^−−^ turnover rates determined with δ^18^O_NO2−_ (*F*_B_) differed somewhat from the *F*_*Ntrace*_ results (Table 3), the congruence can be considered to be adequate because very plausible ranges for NO_2_^−^ turnover rates were observed. Both methods provide a similar range of values, however, the *Ntrace* model does not reflect the significant difference in turnover rates between L1 and L2 (Table 3). This may be due to lower sensitivity of the *Ntrace* approach in precise NO_2_^−^ fluxes determination, since these are determined as a result of complex modelling of all N pools and the final result is an average of the best fit fluxes for both treatments (^15^NH_4_^+^ and ^15^NO_3_^−^). This turnover rate estimation provides a unique opportunity to predicting process rates based on natural abundance isotopic measurements.

Summing up, the natural abundance signatures of NO_2_^−^ can be applied for identification of NO_2_^−^ sources by applying the NA isotope model, which allows to estimate the contribution of the main pathways: NAR, AOX and ORG. This study showed that these are in a very good agreement with the results provided by the *Ntrace* model. Moreover, analysis of δ^18^O_NO2−_ values allows for estimation of NO_2_^−^ turnover rates. The natural abundance signatures of NO_2_^−^ may potentially be used in linking the soil N transformations with gaseous emissions in the form of N_2_O. However, this connection still cannot be fully understood and needs further studies.

## Methods

### Laboratory incubations

#### Oxic incubations: L1 and L2 experiment

Silt loam soil *Albic Luvisol* from arable cropland of Merklingsen experimental station located near Soest (North Rhine-Westphalia, Germany, 51° 34′ 15.5″ N, 8° 00′ 06.8″ E) was used in the incubations (0.87 silt, 0.11 clay, 0.02 sand). The soil density of intact cores was 1.3 g cm^−3^, pH value 6.8, total C content 0.0130, total N content 0.0016, organic matter content 0.0214, initial NO_3_^−^ content 864 μmol N kg^−1^ dry soil and initial NH_4_^+^ content 50 μmol N kg^−1^ dry soil . The soil, upper 30 cm soil layer, was collected on the 18.01.2018 and the incubation was conducted from 19.02.2018 to 05.03.2018. The soil was air dried and sieved at 4 mm mesh size. Afterwards, the soil was rewetted to achieve a water content equivalent to 60% water-filled pore space (WFPS) and fertilised with 20 mg N per kg soil, added as NaNO_3_ (10 mg N) and NH_4_Cl (10 mg N). Three treatments were prepared: natural abundance (NA), labelled with ^15^N nitrate (^15^NO_3_) and labelled with ^15^N ammonium (^15^NH_4_). For the ^15^NO_3_ treatment, NaNO_3_ solution with 72 atom % ^15^N was added and for the ^15^NH_4_ treatment, NH_4_Cl solution with 63 atom % ^15^N was added. Then soils were thoroughly mixed to obtain homogenous distribution of water and fertilizer and an equivalent of 1.69 kg dry soil was repacked into each incubation column with bulk density of 1.3 g cm^−3^.

For each treatment 14 soil columns were prepared, and half of them received additional water injected on the top of the column (100 mL water added) to prepare two moisture treatments: L1 (61% WFPS) and L2 (72% WFPS). The incubation lasted 12 days. In the meantime, on the 6th day of incubation, water addition on the top of each column was repeated (80 mL water added) to increase the soil moisture in both treatments to ca. 68% WFPS in L1 and ca. 81% WFPS in L2. The strategy of adding water on the top of the column to achieve target water content was necessary to allow mixing and compaction at a suitable (low) water content of the soil and thus to optimise homogeneity of water and fertilizer distribution^3^. The incubation temperature was 20 °C. The columns were continuously flushed with a gas mixture with reduced N_2_ content to increase the measurements sensitivity (2% N_2_ and 21% O_2_ in He^35^) with a flow of 9 mL min^−1^. Gas samples were collected daily into two 12 mL septum-capped Exetainer vials (Labco Limited, Ceredigion, UK) connected to the vents of the incubation columns. Soil samples were collected 5 times during the incubation by sacrificing one incubation column per sampling event, which was then divided into three subsamples (replicate samples of mixed soil).

#### Anoxic incubations: L3 experiment

The same soil was used for the static incubations performed under an anoxic atmosphere (N_2_) in closed, gas-tight vessels, where denitrification products accumulated in the headspace. The incubation was conducted from 13.07.2020 to 15.07.2020. The soil was air dried and sieved at 4 mm mesh size. Afterwards, the soil was rewetted to achieve a water content equivalent to 70% water-filled pore space (WFPS) and fertilised with 100 mg N per kg soil, added as NaNO_3_ using natural *Chile saltpetre* (NaNO_3_, Chili Borium Plus, Prills-Natural origin, supplied by Yara, Dülmen, Germany, *δ*^18^O = 56‰, Δ^17^O = 21.8‰) to prepare 12 incubation soil samples of NA treatment and Na^15^NO_3_ to prepare 6 incubation soil samples of ^15^NO_3_^−^ treatment. The soil was thoroughly mixed to obtain a homogenous distribution of water and fertilizer and an equivalent of 85 g of dry soil was repacked into each incubation jar at bulk densities of 1.3 g cm^−3^. The 0.5 dm^3^ Mason jars were used with airtight rubber seals and with two three-way valves installed in their cover to enable sampling and flushing. The jars were flushed with N_2_ at approximately 500 cm^3^ min^−1^ (STP: 273.15 K, 100 kPa) for 10 min to create anoxic conditions. In 6 NA vessels and three ^15^NO_3_^−^ vessels 50 dm^3^ of headspace N_2_ was replaced with 50 dm^3^ of acetylene to inhibit N_2_O reduction to N_2_. Half of the incubation vessels of each treatment was incubated for 45 h and the other half was finished after 21 h for destructive sampling for soil mineral N analyses. The incubation temperature was 20 °C. Four gas samples were collected in 10 to 12 h-intervals by transferring 30 cm^3^ of headspace gases into two pre-evacuated 12 cm^3^ Exetainer vials (Labco Limited, Ceredigion, UK). The excess 3 cm^3^ of headspace gas in each vial ensured that no ambient air entered the vials. The removed sample volume was immediately replaced by pure N_2_ gas.

### Soil analyses

All soil samples were homogenized. Soil water content was determined by weight loss after 24 h drying at 110 °C. Soil pH was determined in 0.01 mol CaCl_2_ solution (ratio 1:5). Nitrate and ammonium concentrations were determined by extraction in 2 M KCl in 1:4 ratio by 1 h shaking. Nitrite concentration was determined in alkaline extraction solution of 2 M KCl with addition of 2 M KOH (25 mL per L) in 1:1 ratio for 1 min of intensive shaking^36^. The amount of added KOH was adjusted to keep the alkaline conditions in extracts (pH over 8). After shaking, the samples were centrifuged for 5 min and filtered. The extracts for NO_2_^−^ measurements were stored at − 4 °C and analyzed within 5 days. NO_3_^−^, NH_4_^+^ and NO_2_^−^ concentrations were determined colorimetrically with an automated analyser (Skalar Analytical B.V., Breda, the Netherlands).

To determine isotopic signatures of mineral nitrogen in NA treatments, microbial analytical methods were applied. For nitrate, the bacterial denitrification method with *Pseudomonas aureofaciens* was applied^37,38^. For nitrite, the bacterial denitrification method for selective nitrite reduction with *Stenotrophomonas nitritireducens* was applied^6^, also for ^15^N -enriched samples from ^15^N treatments. For ammonium, a chemical conversion to nitrite with hypobromite oxidation^39^ followed by bacterial conversion of nitrite after pH adjustment was applied^40^. δ^15^N of the organic N was analysed in the flushed and dried soil sample after mineral N extractions by EA combustion coupled to Delta Plus mass spectrometer (Thermo Finnigan, Bremen, Germany).

In ^15^N treatments, ^15^N abundances of NO_3_^−^ (*a*_NO3−_) and NH_4_^+^ (*a*_NH4+_) were measured as described in Eschenbach, et al.^41^. NO_3_^−^ was reduced to NO by Vanadium-III chloride (VCl_3_) and NH_4_^+^ was oxidized to N_2_ by hypobromite (NaOBr). NO and N_2_ were used as measurement gas. Measurements were performed on isotope ratio mass spectrometer (Delta Plus, Thermo Finnigan, Bremen, Germany).

Soil water was extracted with the method described by Königer, et al.^42^ and the *δ*^18^O of water samples (with respect to VSMOW) was measured using cavity ringdown spectrometer Picarro L1115-*i* (Picarro Inc., Santa Clara, USA). The measurement repeatability (1*σ*) of the internal standards (three calibrated waters with known *δ*^18^O: − 19.67‰, − 8.60‰, + 1.37‰) was below 0.1‰. The overall error associated with the soil water extraction method determined as standard deviation (1*σ*) of the 5 samples replicates was below 0.5‰.

All isotopic values are expressed as ‰ deviation from the ^15^N/^14^N and ^18^O/^16^O ratios of the reference materials (i.e. atmospheric N_2_ and Vienna Standard Mean Ocean Water (VSMOW), respectively).

### Gas analyses

The samples for gas concentration analyses were collected in Exetainer vials (Labco Limited, Ceredigion, UK) and were analysed using an Agilent 7890A gas chromatograph (GC) (Agilent Technologies, Santa Clara, CA, USA) equipped with an electron capture detector (ECD). Measurement repeatability as given by the relative standard deviation (1*σ*) of four standard gas mixtures was typically 1.5%.

The gas samples collected from ^15^N treatments were analyzed for *a*^15^N_N2O_ (^15^N abundance in the emitted N_2_O), *a*_P_N2O_ (^15^N abundance in the ^15^N-pool derived N_2_O) and *f*_P_N2O_ (^15^N-pool derived fraction of N_2_O)^15^ with a modified GasBench II preparation system coupled to MAT 253 isotope ratio mass spectrometer (Thermo Scientific, Bremen, Germany) according to Lewicka-Szczebak et al.^43^. In this set-up, N_2_O is converted to N_2_ during in-line reduction, and stable isotope ratios ^29^R (^29^N_2_/^28^N_2_) and ^30^R (^30^N_2_/^29^N_2_), of N_2_ are determined.

The gas samples of the NA treatment were analysed for N_2_O isotopocules (δ^15^N_N2O_, δ^18^O_N2O_, δ^15^N^SP^_N2O_) using a Delta V isotope ratio mass spectrometer (Thermo Scientific, Bremen, Germany), coupled to an automatic preparation system with Precon + Trace GC Isolink (Thermo Scientific, Bremen, Germany), where N_2_O was pre-concentrated, separated and purified, and m/z 44, 45, and 46 of the intact N_2_O^+^ ions as well as m/z 30 and 31 of NO^+^ fragment ions were determined. The results were evaluated accordingly^44–46^ which allows the determination of average *δ*^15^N, *δ*^15^N^α^ (*δ*^15^N of the central N position of the N_2_O molecule), and *δ*^18^O. *δ*^15^N^β^ (*δ*^15^N of the peripheral N position of the N_2_O molecule) was calculated as *δ*^15^N = ( *δ*^15^N^α^ + *δ*^15^N^β^)/2 and ^15^N site preference (*δ*^15^N^SP^) as *δ*^15^N^SP^ = *δ*^15^N^α^—*δ*^15^N^β^.

### Determination of Δ^17^O excess in N_2_O and NO_3_^−^ and estimation of O-atoms exchange (***x***)

N_2_O samples collected in the L3 NA treatment and N_2_O produced from soil NO_3_^−^ by the bacterial denitrifier method were analysed for *Δ*^17^O after microwave equilibration in a sapphire tube and separation of N_2_ and O_2_ on a mole sieve column^47^. The ^17^O excess*, Δ*^17^O*,* is defined as^48^:

4 $$ \Delta^{17} {\text{O}} = \frac{{{1} + \delta^{{{17}}} {\text{O}}}}{{{(1} + \delta^{{{18}}} {\text{O)}}^{{{0}{\text{.5279}}}} }} - 1 $$

The measurement repeatability (1*σ*) of the international standards (USGS34, USGS35) was typically 0.5‰ for *Δ*^17^O.

The extent of isotope exchange (*x*) was determined based on the comparison of *Δ*^17^O in soil nitrate and produced N_2_O. It requires the application of nitrate characterised by high *Δ*^17^O. Therefore, for this determination, soils in L3 were amended with natural NaNO_3_ *Chile saltpetre* showing high *Δ*^17^O (of 21.8‰) and the *Δ*^17^O of the N_2_O product was measured. *Δ*^17^O of soil water was assumed to be 0‰.

The magnitude of oxygen isotope exchange (*x*) was calculated as:

5 $$ x = 1 - \frac{{\Delta^{17} {\text{O}}({\text{N}}_{{2}} {\text{O}})}}{{\Delta^{17} {\text{O}}({\text{NO}}_{3}^{-} )}} $$

The accuracy of *x* determination was better than 1%.

### Application of the Keeling plot

The original idea for Keeling plot application applies for mixing of the background low level (atmospheric CO_2_) and one dominant source responsible for the significant increase of the CO_2_ concentration^16^. In such a case, plotting the δ values against the reciprocal CO_2_ concentration reveals the isotopic signature of the dominant as intercept of the linear fit^16^. Afterwards, the application of Keeling approach to isotopic studies has expanded to the other environments and substances, including nitrates source identification^17,18,49^. In these studies the requirement of only two sources is not necessarily fulfilled, but the occurrence of a clear linear relation between isotopic signature and reciprocal concentration of the studied substance indicates that there is a dominant source which can be isotopically characterised^49^. This is clearly the case for our nitrite samples, where we find a very significant linear relation (Fig. 1A). Nitrite contents in soils are typically very low and only rarely accumulate, mostly as a result of intensified nitrification or denitrification processes^11,12,49–51^. Hence, with Keeling plot we can isotopically identify the dominant NO_2_^−^ source and identify the pathway responsible for this accumulation.

### Isotope fractionation factors for the nitrite model

The isotope fractionation factors are always expressed as:

$$ \varepsilon_{{{\text{product}}/{\text{substrate}}}} = \, \delta_{{{\text{product}}}} - \, \delta_{{{\text{substrate}}}} $$

Hence, negative ε values inform about normal isotope effect resulting in product depletion in heavy isotopes.

### Nitrite sources

*NAR* is associated with quite high isotopic fractionation of N and O, resulting in significant depletion in ^15^N and ^18^O in the product NO_2_^−^. The nitrate reductase enzymatic experiments showed a mean ^15^ε_NAR_ of − 26.6 ± 0.2‰, similar to ^18^ε_DEN_ with a mean of − 24.9 ± 0.3‰^25^. In pure culture bacterial studies much larger variations of ^15^ε_DEN_ were observed, i.e. ranging from − 30.5 to − 5.4‰^22,24,26^ and it has been suggested that the range from − 15 to − 10‰ is most representative for typical cellular nitrate reduction rates for bacterial strains^27^. The strongest fractionation was found for pure culture fungal studies with a mean ^15^ε_NAR_ of − 37.8 ± 6.6‰^20^. Similar values were found for ^18^ε_DEN_ in pure culture studies: ranging between − 30 and − 25‰ for bacterial denitrification^23^ and between − 30 and − 10‰ for fungal denitrification^20^ . In the sediment denitrification experiments ^15^ε_DEN_ ranged from − 24.4 to − 18.9‰ and ^18^ε_DEN_ from − 21.9 to − 15.8‰^8,29^. A slightly lower ^15^ε_DEN_ of − 29.4 ± 2.4‰ was determined for soil studies^28^.

Nitrite produced from *AOX* is depleted in ^15^N compared to its ammonium substrate. Bacterial ammonia oxidation show a mean ^15^ε_AOX_ of − 25.8 ± 9.8‰^52^, similar to archaeal ammonia oxidation with a mean ^15^ε_AOX_ of − 22 ± 5‰^53^. δ^18^O_NO2-_ from AOX depends on δ^18^O_O2_ (+ 23.5‰), δ^18^O_H2O_ (− 5‰) and δ^18^O_N2Oeq_ (8.6‰) according to the equation^7,54^:

6 $${\delta }^{18}{O}_{AOX}=0.5*\left({\delta }^{18}{O}_{O2}+{\delta }^{18}{O}_{H2O}+20\right)*0.92+\left({\delta }^{18}{O}_{H2O}+{\delta }^{18}{O}_{NO2-eq}\right)*0.08$$

Nitrite produced from *ORG* show much lower ^15^N enrichment with a mean ^15^ε_ORG_ of about − 2‰ as measured for marine sediments fractionation^55^. δ^18^O_NO2−_ from *ORG* was assumed to be the same as for *AOX* according to Eq. (3) (+ 18.4‰).

### Nitrite sinks

Two major nitrite sinks—reduction and oxidation—show opposite isotopic fractionation. Nitrite reduction is associated with normal isotope effect resulting in enrichment in ^15^N and ^18^O of the nitrite pool, whereas nitrite oxidation is characterised by inverse isotope effect, where heavy isotopes are preferentially transferred to the oxidised product leaving nitrite pool depleted in ^15^N and ^18^O^7^. For NIR different fractionation may be associated with various nitrite reductases involved, showing a ^15^ε_NIR_ of − 22 ± 2‰ and an ^18^ε_NIR_ of − 2 ± 2‰ for Cu-NIR and − 8 ± 2‰ and − 6 ± 2‰ respectively for Fe-NIR^56^. In batch experiments with environmental bacterial communities a ^15^ε_DNIR_ ranging from − 15 to − 10‰ was observed when nitrite was investigated as an intermediate product but much lower when nitrite was a substrate^29^. Here we probably also observe this for L3—where nitrite is accumulating we get the best fit with the measured values when no fractionation associated with NIR is assumed (Table 2).

For nitrite oxidation the inverse isotope effects with a ^15^ε_NOX_ of + 12.8^57^ and an ^18^ε_NOX_ of + 5‰^30^ were found.

### Nitrite equilibration with water

The oxygen isotope signature of NO_2_^−^ is additionally modified by the abiotic equilibrium exchange with ambient water^23^. The magnitude of this exchange is governed by the equilibrium isotope effect between NO_2_^−^ and water (ε_eq_) which is a function of temperature^7,23^ and the extend of O atoms exchange. ε_eq_ for the incubation temperature of 20 °C equals 13.63, δ^18^O_H2O_ is − 5‰, consequently, the δ^18^O of nitrite in complete equilibrium with water is 8.6‰. The extend of O atoms exchange was determined with the ^17^O approach^21^ for N_2_O originating for denitrification processes in anoxic experiment L3 and equalled 0.25.

## Supplementary Information

Supplementary information.

## Data Availability

Original data are available upon request. Material necessary for this study findings is presented in the paper and supplementary materials.
