# Supplementary material for: Nitrite isotope characteristics and associated soil N transformations
Source: Sci Rep. 2021 Mar 3;11:5008. doi: 10.1038/s41598-021-83786-w (PMC7930258; doi:10.1038/s41598-021-83786-w)
Supplement: Supplementary file 1 — Supplementary information. [file 41598_2021_83786_MOESM1_ESM.pdf]

Supplement to:

Nitrite isotope characteristics and associated soil N transformations

Dominika Lewicka-Szczebak[1,2], Anne Jansen-Willems[3], Christoph Müller[3,4], Jens Dyckmans[1], Reinhard Well[5]

[1] Centre for Stable Isotope Research and Analysis, University of Göttingen, Büsgenweg 2, 37077 Göttingen, Germany
[2] Laboratory of Isotope Geology and Geoecology, Institute of Geological Sciences, University of Wrocław, Poland
[3] Institute of Plant Ecology, Justus Liebig University, Giessen, Germany
[4] School of Biology and Environmental Science and Earth Institute, University College Dublin, Belfield, Dublin, Ireland
[5] Thünen-Institut of Climate-Smart Agriculture, Bundesallee 50, 38116 Braunschweig, Germany

Correspondence to: Dominika Lewicka-Szczebak (dominika.lewicka-szczebak@uwr.edu.pl)

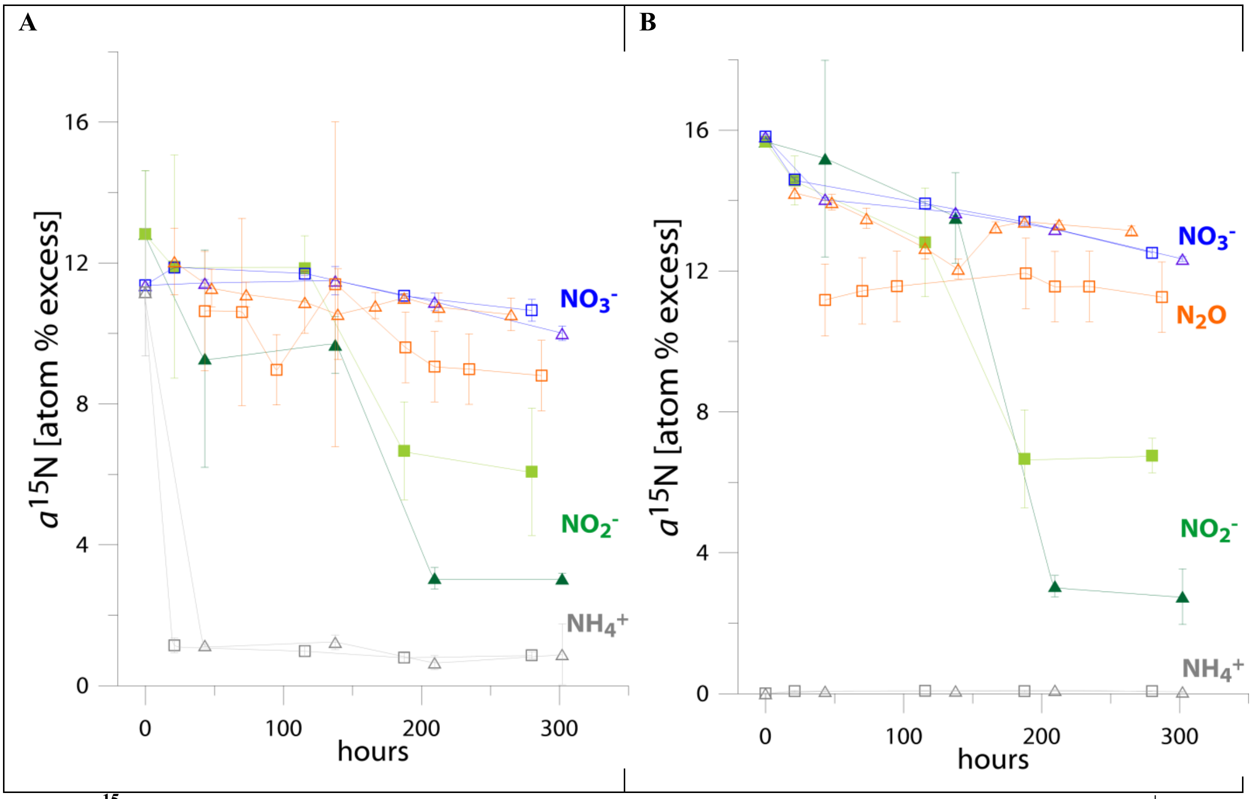

Fig S1 ^{15}**N abundance in soil mineral N compounds: NO_2^- (green points), NO_3^- (blue points), NH_4^+ (grey points), and in produced N_2O (orange points) in L1 (triangles) and L2 (squares) in $^{15}NH_4^+$ (A) and $^{15}NO_3^-$ treatment (B).**

Table S1 Results summary (mean values and standard deviations* for all samplings are presented)

	treatment	L1a	L1b	L2a	L2b	L3
WFPS [%]		61	68	72	81	70
conditions		oxic	oxic	oxic	oxic	anoxic
NO_2 content	NA	0.014 ± 0.003	0.011 ± 0.002	0.028 ± 0.022	0.010 ± 0.005	0.674 ± 0.272
[mg N kg^{-1}	$^{15}NH_4^+$	0.013 ± 0.007	0.007 ± 0.000	0.054 ± 0.051	0.011 ± 0.002	
soil]	$^{15}NO_3^-$	0.024 ± 0.011	0.007 ± 0.001	0.036 ± 0.033	0.013 ± 0.004	0.171 ± 0.048
NO_3 content	NA	20.6 ± 0.7	21.7 ± 1.8	20.6 ± 1.7	21.3 ± 1.8	152.2 ± 7.6
[mg N kg^{-1}	$^{15}NH_4^+$	21.3 ± 0.5	22.1 ± 1.0	19.3 ± 1.9	20.1 ± 2.4	
soil]	$^{15}NO_3^-$	20.8 ± 0.6	21.1 ± 0.7	20.0 ± 0.7	18.9 ± 1.2	149.7 ± 1.5
NH_4 content	NA	0.33 ± 0.05	0.36 ± 0.07	0.40 ± 0.17	0.66 ± 0.26	
[mg N kg^{-1}	$^{15}NH_4^+$	0.67 ± 0.11	0.66 ± 0.12	0.86 ± 0.14	0.90 ± 0.12	
soil]	$^{15}NO_3^-$	0.65 ± 0.07	0.53 ± 0.24	0.73 ± 0.05	0.72 ± 0.20	
$a^{15}N_{NO2}$	$^{15}NH_4^+$	9.8 ± 2.0	3.4 ± 0.2	12.2 ± 2.1	6.7 ± 1.5	
[atom %]	$^{15}NO_3^-$	14.7 ± 2.2	3.2 ± 0.6	14.1 ± 1.4	7.1 ± 0.9	0.41 ± 0.01
$a^{15}N_{NO3}$	$^{15}NH_4^+$	11.8 ± 0.3	10.8 ± 0.5	12.2 ± 0.1	11.2 ± 0.3	
[atom %]	$^{15}NO_3^-$	14.2 ± 0.2	13.1 ± 0.5	14.6 ± 0.4	13.3 ± 0.5	0.42 ± 0.01
$a^{15}N_{NH4}$	$^{15}NH_4^+$	1.6 ± 0.1	1.4 ± 0.4	1.5 ± 0.2	1.2 ± 0.1	
[atom %]	$^{15}NO_3^-$	0.5 ± 0.0	0.5 ± 0.0	0.5 ± 0.0	0.5 ± 0.0	
$a^{15}N_{N2O}$	$^{15}NH_4^+$	10.4 ± 1.0	9.1 ± 0.3	11.2 ± 0.6	10.8 ± 0.2	
[atom %]	$^{15}NO_3^-$	11.6 ± 0.3	11.5 ± 0.2	13.1 ± 0.7	13.3 ± 0.1	0.40 ± 0.00
a_{P_N2O}	$^{15}NH_4^+$	16.8 ± 5.9	12.0 ± 0.9	14.0 ± 2.5	11.1 ± 0.2	
[atom %]	$^{15}NO_3^-$	17.0 ± 2.4	18.2 ± 0.9	15.2 ± 1.2	13.5 ± 0.2	0.40 ± 0.00
f_{P_N2O} [a]	$^{15}NH_4^+$	0.69 ± 0.09	0.81 ± 0.06	0.86 ± 0.12	1.00 ± 0.03	1.00 ± 0.00
	$^{15}NO_3^-$	0.70 ± 0.07	0.66 ± 0.04	0.92 ± 0.09	1.00 ± 0.00	
$\delta^{15}N_{NO2}$	NA	-0.6 ± 1.9	6.5 ± 2.0	0.4 ± 5.1	6.5 ± 2.4	-24.8 ± 3.3
$\delta^{18}O_{NO2}$	NA	11.1 ± 3.1	12.5 ± 2.5	14.5 ± 5.5	10.8 ± 4.2	5.5 ± 1.4
$\delta^{15}N_{NO3}$	NA	4.4 ± 0.2	4.7 ± 0.5	4.2 ± 0.1	5.1 ± 0.5	7.0 ± 2.1
$\delta^{18}O_{NO3}$	NA	4.5 ± 0.1	4.1 ± 0.4	4.8 ± 0.2	4.7 ± 0.6	15.3 ± 2
$\delta^{15}N_{NH4}$	NA	88.7 ± 2.4	99.2 ± 1.1	80.8 ± 7	54.0 ± 17.8	
$\delta^{15}N_{N2O\_p}$ [b]	NA	-18.9 ± 3.2	-11.3 ± 9.6	-18.7 ± 3.5	-36.7±5.1	-45.9 ± 1.4
$\delta^{18}O_{N2O\_p}$ [b]	NA	9.9 ± 5.7	2.1 ± 6.6	20.2 ± 3.1	26.6 ± 3.3	12.6 ± 1.0
$\delta^{15}N^{SP}_{N2O\_p}$ [b]	NA	-3.8 ± 5.0	-3.4 ± 8.4	0.4 ± 3.0	9.8 ± 1.3	-2.9 ± 1.1

* 1σ of four replicates and 5 samplings

[a] ^{15}N-pool derived fraction (for L1 and L2 published in [1])

[b] isotopic signature of the produced N_2O (corrected for the N_2O reduction effect, based on the values published for L1 and L2 in [1])

Table S2 Results summary of the *Ntrace* model based on the ^{15}N traced traetments

***Ntrace* turnower rates [µg kg^{-1} d^{-1}]**						
	treatment	L1a	L1b	L2a	L2b	
Ntrace*	here	1100 ± 0	3392 ± 0	1097 ± 0	3380 ± 0	
Mnrec		646 ± 18	3036 ± 126	813 ± 59	2966 ± 179	mineralisation of recalcitrant Norg to NH_4
INH4-Nrec		0 ± 0	0 ± 0	0 ± 0	0 ± 0	immoblization NH_4 to recalcitrant Norg
Mnlab		525 ± 0	212 ± 0	284 ± 0	211 ± 0	mineralisation of labile Norg to NH_4
INH4-Nlab		0 ± 0	30 ± 26	4 ± 3	27 ± 23	immoblization NH_4 to labile Norg
Onrec		250 ± 60	2 ± 1	35 ± 14	6 ± 6	oxidation Nrec to NO_3
INO3-Nrec		1157 ± 0	1006 ± 0	307 ± 0	304 ± 0	immobilization of NO_3 to recalcitrant Norg
Onlab		7 ± 0	92 ± 0	7 ± 0	90 ± 0	oxidation Nlab to NO_3
INO3-Nlab		26 ± 7	108 ± 36	33 ± 14	60 ± 35	immobilization of NO_3 to labile Norg
ONH4	AOX	4 ± 0	13 ± 0	4 ± 0	13 ± 0	oxidation of NH_4 to NO_2
DNO3		34 ± 0	208 ± 0	18 ± 0	208 ± 0	dissimilatory NO_3 reduction to NH_4
ANH4		5 ± 0	49 ± 0	6 ± 0	57 ± 0	adsorption NH_4
RNH4a		10 ± 0	186 ± 0	10 ± 0	182 ± 0	realease of adsorbed NH_4
A_NO3		5 ± 0	0 ± 0	5 ± 0	0 ± 0	adsortion NO_3
R_NO3s		108 ± 36	152 ± 47	2 ± 2	53 ± 53	realease of stored NO_3
ONO2n	NIOX	21 ± 8	19 ± 13	286 ± 141	147 ± 64	oxidation NO_2nit NO_3
ONorg	ORG	10 ± 5	1 ± 0	3 ± 2	45 ± 47	oxidation of Nrec to NO_2org
ONO2o	NIOX	145 ± 51	171 ± 67	270 ± 64	233 ± 104	oxidation of NO_2org to NO_3
RNO3	NAR	2 ± 0	6 ± 3	30 ± 21	6 ± 5	reduction of NO_3 to NO_2den
RNO2n	NIR	150 ± 52	203 ± 72	271 ± 63	236 ± 104	reduction of NO_2nit to Ngas
RNO2d	NIR	11 ± 6	26 ± 9	281 ± 111	101 ± 56	reduction of NO_2den to Ngas
RNO2o	NIR	1100 ± 0	3392 ± 0	1097 ± 0	3380 ± 0	reduction of NO_2org to Ngas
***Ntrace* fractions**						
NO_2^- sources	NAR	0.75 ± 0.26	0.46 ± 0.11	0.57 ± 0.22	0.53 ± 0.24	reduction of NO_3 to NO_2den
	AOX	0.14 ± 0.04	0.06 ± 0.02	0.36 ± 0.12	0.14 ± 0.08	oxidation of NH_4 to NO_2
	ORG	0.11 ± 0.04	0.49 ± 0.24	0.07 ± 0.04	0.33 ± 0.15	oxidation of Nrec to NO_2org
NO_2^- sinks	NIR	0.58 ± 0.07	0.99 ± 0.11	0.61 ± 0.07	0.78 ± 0.12	reduction of NO_2 to Ngas
	NIOX	0.42 ± 0.07	0.01 ± 0.00	0.39 ± 0.06	0.22 ± 0.11	oxidation of NO_2 to NO_3
NH_4^+ sources	Mnrec	0.99 ± 0.00	0.99 ± 0.00	0.98 ± 0.00	0.98 ± 0.00	mineralisation of Nrec
	Mnlab	0.00 ± 0.00	0.00 ± 0.00	0.00 ± 0.00	0.00 ± 0.00	mineralisation of Nlab
	DNO3	0.00 ± 0.00	0.00 ± 0.00	0.00 ± 0.00	0.00 ± 0.00	dissimilatory NO_3 reduction to NH_4
	RNH4a	0.01 ± 0.00	0.01 ± 0.00	0.01 ± 0.00	0.02 ± 0.00	realease of adsorbed NH_4
NH_4^+ sinks	INH4_Nrec	0.52 ± 0.02	0.71 ± 0.05	0.85 ± 0.10	0.86 ± 0.15	immoblization of NH_4 to Nrec
	INH4_Nlab	0.43 ± 0.00	0.25 ± 0.00	0.06 ± 0.00	0.06 ± 0.00	immoblization of NH_4 to Nlab
	AOX	0.02 ± 0.01	0.03 ± 0.01	0.03 ± 0.03	0.02 ± 0.00	oxidation of NH_4 to NO_2
	ANH4	0.03 ± 0.00	0.02 ± 0.00	0.06 ± 0.00	0.06 ± 0.00	adsorption NH_4
NO_3^- sources	On	0.99 ± 0.00	0.97 ± 0.00	1.00 ± 0.02	0.88 ± 0.02	oxidation Norg to NO_3
	R_NO3s	0.00 ± 0.00	0.00 ± 0.00	0.02 ± 0.00	0.00 ± 0.00	realease of stored NO_3
	ONO2o	0.01 ± 0.00	0.00 ± 0.00	0.01 ± 0.00	0.12 ± 0.04	oxidation of NO_2org to NO_3-
NO_3^- sinks	INO3	0.62 ± 0.15	0.13 ± 0.03	0.20 ± 0.00	0.18 ± 0.01	immobilization of NO_3
	DNO3	0.01 ± 0.00	0.01 ± 0.00	0.03 ± 0.00	0.03 ± 0.00	dissimilatory NO_3 reduction to NH_4
	A_NO3	0.02 ± 0.00	0.03 ± 0.00	0.40 ± 0.00	0.35 ± 0.00	adsortion NO_3
	NAR	0.35 ± 0.12	0.82 ± 0.15	0.37 ± 0.16	0.44 ± 0.25	reduction of NO_3 to NO_2den

*Abbreviations for N transformation processes according to [2]

Table S3 Nitrite stable isotope model for dry (L1a, L2a) and wet (L1b, L2b) parts of the L1 and L2 experiments. Nitrite isotopic signature ($\delta^{18}O$, $\delta^{15}N$) is modelled based on: (i) the nitrite sources taking into account measured substrate isotopic signatures (NO_3^- for *NAR*, NH_4^+ for *AOX* and organic N for *ORG*), fractionation factors ($^{18}\varepsilon$, $^{15}\varepsilon$), and sources mixing proportions according to the results of the N_{trace} model (f_{mix} N_{trace}); (ii) nitrite sinks with their characteristic isotopic fractionation factors ($^{18}\varepsilon$, $^{15}\varepsilon$) including the nitrite reduction-oxidation ratio after results of the N_{trace} model; and (iii) nitrite equilibration with water (NO_2^- eq) including measured extend of O-exchange of 0.25, $\delta^{18}O$ of -5‰ and $^{18}\varepsilon_{eq}$ for 20°C. The model results were fitted to the measured values by adjusting the sources mixing proportions (f_{mix} *fitted*) to find the ideal fit of modelled *vs.* measured $\delta^{15}N$ values.

	source	substrate $\delta^{18}O$	$\delta^{15}N$	produced NO_2^- $\delta^{18}O$	$\delta^{15}N$	f_{mix} N_{trace}	mixed NO_2^- $\delta^{18}O$	$\delta^{15}N$	sink	residual NO_2^- $\delta^{18}O$	$\delta^{15}N$	NO_2^- eq $\delta^{18}O$	$f_{red\text{-}ox}$ N_{trace}	final modeled $\delta^{18}O$	$\delta^{15}N$	true measured $\delta^{18}O$	$\delta^{15}N$	f_{mix} *fitted*
**L1a**	*NAR*	4.5	4.4	4.5	-13.4	**0.75**	7.9	-0.8	*NIR*	11.9	9.2	11.1	0.58	8.3	-0.5	11.1	-0.6	**0.75**
	AOX	23.5; -6.4	88.7	18.4	63.7	**0.14**			*NIOX*	2.9	-13.8	4.3	0.42					**0.13**
	ORG	23.5	7.4	18.4	5.4	**0.11**												**0.11**
**L1b**	*NAR*	4.1	4.7	4.1	-13.1	**0.46**	11.9	0.8	*NIR*	15.9	10.8	14.1	0.99	14.0	10.6	12.5	6.5	**0.48**
	AOX	23.5; -6.4	99.2	18.4	74.2	**0.06**			*NIOX*	6.9	-12.2	7.3	0.01					**0.00**
	ORG	23.5	7.4	18.4	5.4	**0.49**												**0.52**
**L2a**	*NAR*	4.8	4.2	4.8	-13.6	**0.57**	10.6	12.7	*NIR*	14.6	22.7	13.1	0.61	10.4	13.7	14.5	0.4	**0.66**
	AOX	23.5; -6.4	80.8	18.4	55.8	**0.36**			*NIOX*	5.6	-0.3	6.4	0.39					**0.13**
	ORG	23.5	7.4	18.4	5.4	**0.07**												**0.21**
**L2b**	*NAR*	4.7	5.1	4.7	-12.7	**0.53**	11.1	-1.0	*NIR*	15.1	9.0	13.5	0.78	12.0	3.9	10.8	6.5	**0.48**
	AOX	23.5; -6.4	54.0	18.4	29.0	**0.14**			*NIOX*	6.1	-14.0	6.8	0.22					**0.21**
	ORG	23.5	7.4	18.4	5.4	**0.33**												**0.31**

References

1 Lewicka-Szczebak, D., Lewicki, M. P. & Well, R. N_2O isotope approaches for source partitioning of N_2O production and estimation of N_2O reduction – validation with ^{15}N gas-flux method in laboratory and field studies. *Biogeosciences Discussions*, doi:https://doi.org/10.5194/bg-2020-209 (2020).

2 Müller, C., Laughlin, R. J., Spott, O. & Rütting, T. Quantification of N_2O emission pathways via a ^{15}N tracing model. *Soil Biol Biochem* **72**, 44-54 (2014).
